# Tórax inestable y mediastinitis con pérdida esternal total poscirugía cardíaca pediátrica. Técnica de reconstrucción y reporte de caso

**DOI:** 10.47487/apcyccv.v4i4.326

**Published:** 2023-12-27

**Authors:** Luis J. Palma-Ortecho, Henry Peralta-Santos, Tommy L. Prado-Gómez

**Affiliations:** 1 Servicio de Cirugía Cardiovascular Pediátrica, Instituto Nacional Cardiovascular (INCOR), Lima, Perú. Servicio de Cirugía Cardiovascular Pediátrica Instituto Nacional Cardiovascular (INCOR) Lima Perú; 2 Servicio de Cuidados Intensivos Pediátricos, Instituto Nacional Cardiovascular (INCOR), Lima, Perú. Servicio de Cuidados Intensivos Pediátricos Instituto Nacional Cardiovascular (INCOR) Lima Perú

**Keywords:** Tórax Inestable, Mediastinitis, Toracoplastia, Flail Chest, Mediastinitis, Thoracoplasty

## Abstract

Se presenta el caso de una niña de 2 años de edad, con antecedente de una cirugía de *banding* pulmonar, a quien se le realiza una técnica de estabilización de pared torácica con barras de titanio y cobertura con *flaps* musculares por presentar mediastinitis posquirúrgica asociada a pérdida esternal total, luego de una cirugía de cierre de comunicación interventricular, *debanding* y plastia de arteria pulmonar. La paciente presentó evolución posquirúrgica favorable.

## Abstract (en)

We present the case of a two-year-old girl, with a history of pulmonary banding surgery who underwent a chest wall stabilization technique with titanium rods and muscle flaps coverage, due to post-surgical mediastinitis associated with total sternal loss after ventricular septal defect closure surgery, debanding, and pulmonary artery plasty. The patient had a favorable postsurgical evolution.

## Introducción

La infección de sitio quirúrgico profundo conocida como infección posesternotomía del espacio mediastínico o mediastinitis, es una complicación grave de la cirugía cardíaca que puede estar asociada a osteomielitis esternal ^1^^,^^2^. Tiene una incidencia de entre 0,2 al 5% ^3^, con una tasa de mortalidad de hasta 40% ^4^. En cirugía cardiaca pediátrica se han reportado una incidencia entre 1,7 a 8,0 por 100 casos ^1^.

Como factores de riesgo identificados se tiene a la edad menor de 1 mes, síndromes genéticos, hospitalización previa mayor de 48 h, uso de hipotermia intraoperatoria, necesidad de múltiples procedimientos durante la misma cirugía, duración de la cirugía y presencia de hilos de marcapaso temporales por más de 3 días ^1^. El cierre esternal diferido podría incrementar el riesgo de infecciones siendo la tasa muy variable entre 1% al 28% ^5^.

Existen múltiples procedimientos quirúrgicos y materiales que se utilizan en la población adulta para el tratamiento de mediastinitis y reconstrucción de la pared torácica, como mallas y parches (politetrafluoroetileno, biológicos), prótesis rígidas y semirrígidas (aloinjertos o xenoinjertos de hueso), titanio, cerámica de calcio, prótesis con impresión tridimensional, polímeros biodegradables, nanocompuestos e injertos óseos derivados de células madre ^1^; sin embargo, en la población pediátrica existe poca información sobre ellos.

La reconstrucción de la pared torácica sigue siendo un desafío, más aún en niños, donde no se disponen de materiales adecuados para la edad y peso; muchas veces se requiere de un abordaje multidisciplinario, incluyendo al cirujano cardiotorácico, cirujano plástico, etc. Siendo objetivos de la reconstrucción el restaurar la rigidez de la pared torácica, preservar la mecánica pulmonar y proteger los órganos intratorácicos; minimizando la deformidad torácica ^6^.

Presentamos un caso de tratamiento quirúrgico exitoso en una niña con mediastinitis posquirúrgica, asociada a pérdida esternal total y tórax inestable, luego de una cirugía de cierre de comunicación interventricular (CIV) y *debanding* de arteria pulmonar realizada por el equipo de cirugía cardiopediátrica de nuestra institución.

## Reporte de caso

Se trata de una niña de 2 años de edad, 14,2 kg de peso, con diagnóstico de comunicación interventricular y con el antecedente de una cirugía de *banding* de la arteria pulmonar, realizada al año de edad por abordaje medioesternal.

En nuestra institución y de manera electiva, la paciente fue sometida a cirugía cardiaca para corrección de defectos. Por abordaje medioesternal, canulación central aortobicaval, en hipotermia leve, se realizó cierre de comunicación interventricular con parche de pericardio bovino, *debanding* y plastía de arteria pulmonar con un tiempo de circulación extracorpórea (CEC) prolongado. El cierre esternal se realizó con refuerzo del borde esternal izquierdo tipo Robicsek y paciente fue trasladada a la unidad de cuidado intensivo (UCI) pediátrico.

Al tercer día posoperatorio, la paciente es reintervenida quirúrgicamente por presentar estenosis severa en el origen de ambas ramas pulmonares, se realizó plastía de arteria y ramas pulmonares con parche de pericardio bovino.

Siete días después la paciente reingresó a sala de operaciones por dehiscencia esternal e infección de sitio operatorio y alta sospecha de mediastinitis. Entre los hallazgos intraoperatorios se describen: infección y dehiscencia de herida operatoria; dehiscencia de sutura esternal en su totalidad; pérdida total de esternón debido a múltiples fracturas en zonas de osteosíntesis previa y compromiso importante de cartílagos condrocostales con necrosis e infección; se encontró también, abundante secreción purulenta en mediastino. Se tomaron distintas muestras para cultivo, anatomía patológica y se procedió a limpieza quirúrgica con desbridamiento extenso de tejidos necróticos; apertura extensa de ambas pleuras; colocación de drenes pleurales; dren mediastínico; colocación de catéter para irrigación mediastinal y cierre de la piel con puntos totales

La mediastinitis se confirmó en sala de operaciones debido al hallazgo de secreción purulenta, en tanto que la anatomía patológica demostró osteomielitis aguda. En la UCI la paciente evolucionó con hemodinamia estable, se brindó soporte ventilatorio por inestabilidad torácica y se dejó irrigación mediastinal con solución fisiológica por 5 días.

**Figure 1:**
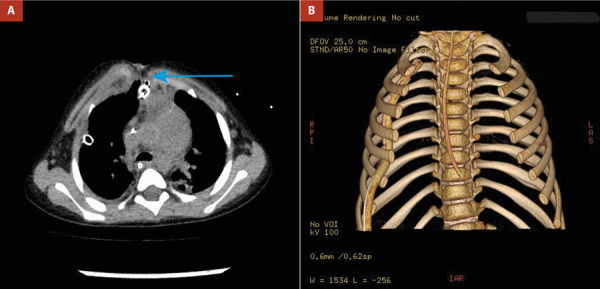
Niña de 02 años de edad con mediastinitis y pérdida total de esternón. **(A)** TEM tórax con evidencia de ausencia de esternón (flecha azul). **(B)** TEM tórax con reconstrucción ósea se observa ausencia esternal.

Pasadas 3 semanas de la última cirugía y con la infección controlada, en junta médica multidisciplinaria se decidió realizar toracoplastia con barras de titanio para la estabilización del tórax, previa realización de TEM torácica con reconstrucción de pared torácica **(**Figura 1**)**.

### Técnica quirúrgica

Se realizó la reapertura de piel a nivel de incisión previa, disección por planos hasta alcanzar cartílagos costales, curetaje de bordes de cartílagos, lavado de cavidad, liberación de colgajo muscular (pectoral mayor bilateral, vaina anterior del músculo recto anterior del abdomen y parte de la aponeurosis del oblicuo externo). 

**Figura 2:**
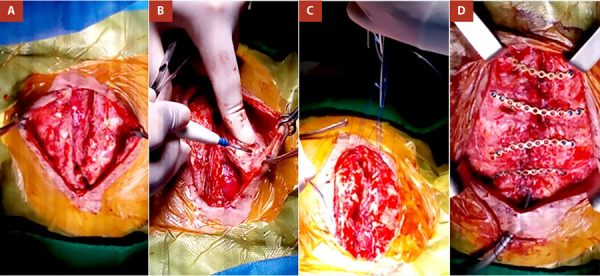
Vista intraoperatoria. **(A)** Apertura de piel y tejido celular subcutáneo, se observa ausencia esternal. **(B)** Liberación de colgajo muscular, pectoral mayor. **(C)** Afronte de cartílagos costales con sutura de nylon 1/0. **(D)** Colocación de 4 miniplacas de titanio.

Se afrontaron cartílagos costales con sutura de Nylon 1/0 **(**Figura 2**)**; se coloca 04 miniplacas de titanio 2,0 en forma de C (CONMET; LLC 24/1 Onezhskaya str. Moscow) adaptadas a las curvas costales en ambos hemitórax, las placas son fijadas a las costillas 1, 2, 4 y 5 con sutura de alambre quirúrgico 1/0 (TAGUM) y 02 minitornillos de titanio 5 mm (CONMET; LLC 24/1 Onezhskaya str. Moscow) por lado. 

**Figura 3:**
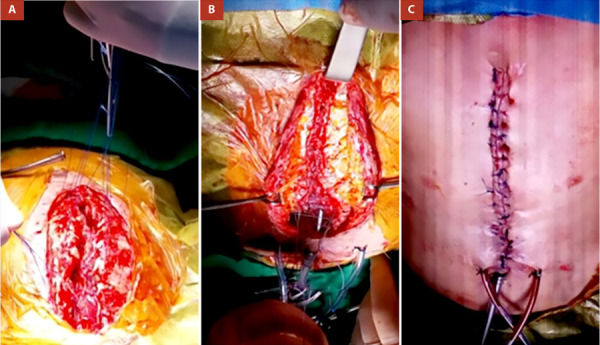
Vista intraoperatoria. **(A)** Afronte de colgajos musculares. **(B)** cobertura de todo el material protésico **(C)** Cierre de piel y tejido celular subcutáneo con sutura de Nylon, drenaje mediastínico y drenajes subpectoral bilateral.

Se colocó 01 dren mediastinal 20 Fr y Hemovac 14 Fr subpectoral, bilateral. Se aproximaron colgajos musculares hacia la línea media con sutura de poliglactina 2/0, logrando cubrir la totalidad de las barras de titanio. El cierre de piel y tejido celular subcutáneo se realizó con puntos totales de Nylon 2/0 **(**Figura 3**)**.

### Evolución posoperatoria

**Figura 4:**
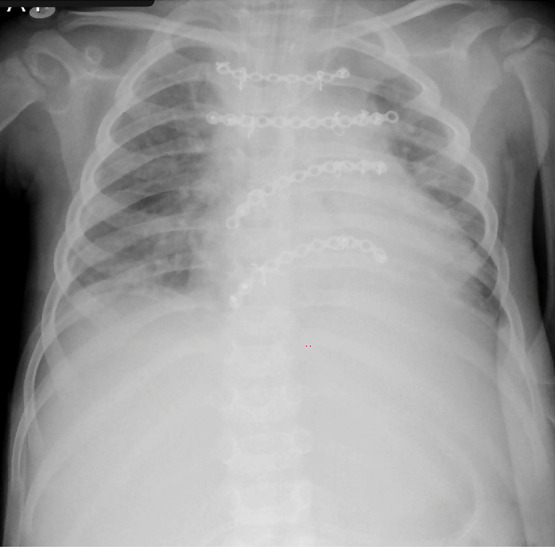
Radiografía de tórax al sexto día posoperatorio luego de plastía torácica, se observa 04 barras de titanio.

Tras la cirugía de plastía torácica **(**Figura 4**)**, la paciente se mantuvo en ventilación mecánica, con sedoanalgesia, sin soporte inotropovasopresor, continuando antibioticoterapia parenteral. Luego de 6 días de ventilación mecánica, con terapia para manejo de síndrome de abstinencia, habiendo recibido corticoides profilácticos, se realizó extubación programada; continuando soporte respiratorio posextubación con CPAP nasal por 6 días, para luego pasar a cánula binasal. Se brindó fisioterapia respiratoria en todo el proceso pre y posextubación. La paciente completó 6 semanas de antibioterapia parenteral y tras 2 meses de estancia hospitalaria fue dada de alta. En el seguimiento a los 8 meses, se encuentra asintomática, sin medicación. 

## Discusión

La mediastinitis es una grave complicación de la cirugía cardiaca que alcanza una mortalidad de hasta el 40% ^7^; la osteomielitis esternal puede ser una complicación infrecuente, pero con una mortalidad reportada entre 7 - 80% ^8^. Los gérmenes aislados con mayor frecuencia son los Gram positivos.

Tanto en adultos como en niños se han descrito muchas técnicas para el tratamiento de una mediastinitis, entre los que se incluyen el desbridamiento quirúrgico extenso; el cierre primario o diferido de esternotomía; la interposición de *flaps* musculares; el colgajo de epiplón mayor y el uso de presión negativa, todos asociados al uso de antibioticoterapia ^7^^-^^9^. Sin embargo, cuando se asocia a pérdida total de esternón, encontramos muchos reportes en pacientes adultos en los que se describe el uso de materiales sintéticos, biológicos y metálicos; además, se asocian tecnologías como la tomografía computarizada con reconstrucción tridimensional de imágenes (3D) que podrían guiar una producción precisa de prótesis esternales a través de una tecnología de impresión 3D. En niños se dispone de limitada experiencia en el uso de estos materiales, por el peso y, sobre todo, por el potencial crecimiento de su anatomía torácica.

La indicación de la reconstrucción de la pared torácica es necesaria para defectos mayores o cuando hay compromiso de la función respiratoria. La principal finalidad es restablecer la integridad de la pared y mantener su estética, así como mejorar la dinámica respiratoria. Las barras de titanio se presentan como una alternativa de uso para este tipo de pacientes ^10^. Existe experiencia en fijación de la pared torácica con barras de titanio en población adulta, con buenos resultados ^11^, lo que no se describe en población pediátrica.

En nuestro medio solo contamos con material de osteosíntesis costal para adultos, siendo todo un desafío planificar la estabilización torácica y el momento adecuado para ello, puesto que nuestra paciente presentó pérdida esternal total debido a la mediastinitis.

Realizando una evaluación de los materiales disponibles en nuestro medio, se planificó y realizó la estabilización torácica con miniplacas de titanio que son de uso rutinario en cirugía maxilofacial, por ser las más compatibles con el diámetro de los cartílagos costales de nuestra paciente; además, las barras de titanio presentan ventajas de flexibilidad y fuerza que permite adecuarlo a la curvatura y angulación de los costillas y puede ser usado sin retirar el periostio permitiendo maximizar la perfusión del hueso ^5^. El uso de colgajos musculares de ambos pectorales mayores permitió cubrir la totalidad de los cartílagos costales y las placas de titanio, lo cual permitió cerrar algún espacio muerto con tejido bien vascularizado.

El tiempo óptimo para la estabilización del tórax aún no está claro, pues algunos autores recomiendan tratamiento y reconstrucción temprana de la pared torácica debido a que prolongar este tiempo no ha demostrado buenos resultados ^12^.

La paciente presentó una adecuada evolución posquirúrgica, con extubación programada a los 6 días de la cirugía de estabilización torácica. Sin embargo, tuvo tiempo de ventilación prolongada previa a la estabilización torácica, primero para el control del proceso infeccioso y luego para disponer de los materiales adecuados a usar.

En conclusión, la estabilización torácica tras la pérdida esternal total posmediastinitis con barras de titanio y movilización de colgajos musculares de pectoral mayor, permitió una adecuada estabilización torácica en nuestra paciente; manteniendo una adecuada dinámica respiratoria tras extubación, integridad y estética de la pared torácica a corto y mediano plazo. Siendo esta técnica quirúrgica oportunidad de investigación en pacientes pediátricos.

## Fuente de financiamiento:

Autofinanciado
